# Case Report: Ultrasound-guided interventional diagnosis and treatment of gastrosplenic fistula caused by lymphoma

**DOI:** 10.3389/fonc.2025.1638866

**Published:** 2025-08-20

**Authors:** Yahan Zhang, Yang Li, Manxi Li, Yu Song

**Affiliations:** Department of Ultrasound, The Second Affiliated Hospital of Dalian Medical University, Dalian, China

**Keywords:** lymphoma, gastrosplenic fistula, ultrasound, interventional, contrast-enhanced ultrasound

## Abstract

Gastrosplenic fistula is a rare complication, most often secondary to gastric or splenic lymphoma. Severe gastrosplenic fistula can cause life-threatening upper gastrointestinal bleeding, making early diagnosis and intervention critical for a favorable prognosis. Currently, surgical intervention remains the primary treatment; however, outcomes are often suboptimal. In this paper, we present a case involving ultrasound-guided interventional diagnosis and treatment of gastrosplenic fistula caused by diffuse large B-cell lymphoma. An 18-year-old male initially presented with gastric distension and epigastric pain and was diagnosed with gastric non-Hodgkin lymphoma (diffuse large B-cell lymphoma) at an outside hospital before being referred to our institution for further management. Contrast-enhanced CT revealed an abnormal density lesion between the gastric fundus and spleen, suggestive of gastric perforation accompanied by adjacent exudation and localized abscess formation. A multidisciplinary team evaluation identified markedly elevated inflammatory markers and poor overall condition, rendering the patient unsuitable for immediate surgery. Subsequent B-mode ultrasound and contrast-enhanced ultrasound (CEUS) precisely delineated the fistula location and extent of the abscess, enabling determination of a safe puncture path. Ultrasound-guided percutaneous catheter drainage of the gastrosplenic fistula was then successfully performed. Post-procedural intracavitary contrast injection confirmed correct catheter tip placement distal to the fistula. Follow-up CT imaging 20 days after drainage showed a significant reduction in the encapsulated fluid and gas collection at the fistula site. After one month of clinical improvement, the patient underwent total gastrectomy with resection of the pancreatic body-tail and spleen. He was subsequently discharged and continued maintenance chemotherapy for non-Hodgkin lymphoma. At 13 months postoperatively, the patient remains clinically stable with normal vital signs.

## 1 Introduction

Gastrosplenic fistula is a rare but potentially life-threatening complication arising from various conditions, including lymphoma, gastric adenocarcinoma, Crohn’s disease, splenic abscess, trauma, and chemotherapy (1, 2). Among these, lymphoma is the most common cause. Surgical intervention remains the primary treatment modality, typically involving open procedures such as fistulectomy, splenectomy, and gastrectomy. CEUS, which employs intravenous microbubble contrast agents, enhances the visualization of vascular structures and arterial perfusion within lesions. This technique is particularly valuable for identifying avascular tissue regions, delineating fistulas, and monitoring abscess formation. Ultrasound-guided, including CEUS-guided, percutaneous drainage has become a widely adopted minimally invasive approach, especially effective in managing cystic lesions and fluid collections. Based on clinical and imaging data, we propose a minimally invasive strategy that combines CEUS with ultrasound-guided percutaneous catheter drainage for the treatment of gastrosplenic fistula. This approach offers a valuable therapeutic window for patients who are not immediate surgical candidates and may provide important insights for managing this rare and complex condition.

## 2 Case presentation

**Figure 1:**
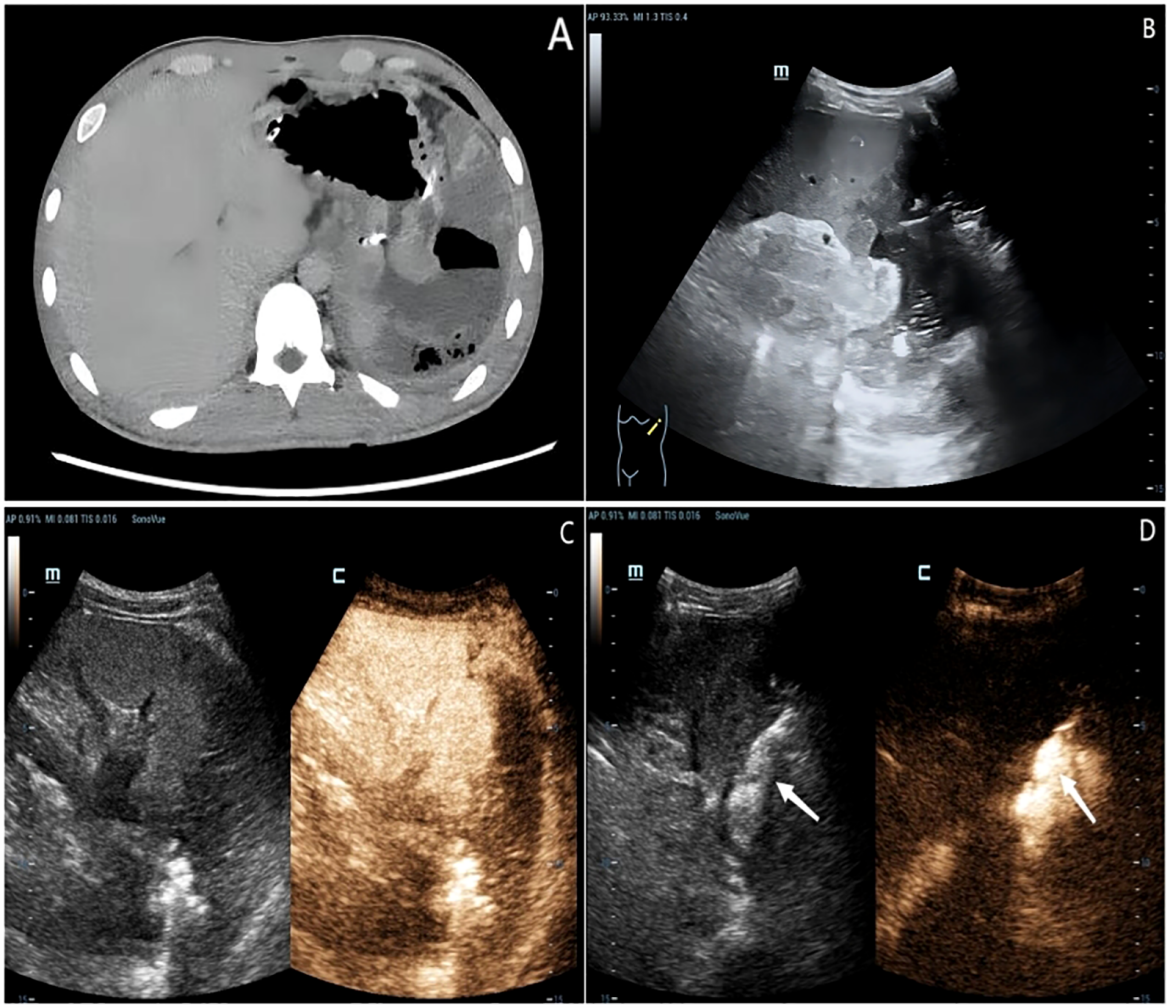
An 18-year-old male patient diagnosed with gastric non-Hodgkin’s lymphoma nearly one month ago. **(A)** Contrast-enhanced CT scan demonstrated an abnormal density lesion between the gastric fundus and spleen. **(B)** B-mode ultrasound revealed heterogeneous echogenicity within the spleen and a large irregular heterogeneous mass adjacent to the splenic hilum containing fluid components and extensive gas-like hyperechoic foci. **(C)** Contrast-enhanced ultrasound identified a large non-enhancement area in the inferolateral spleen during the arterial phase and a fistulous tract connecting the gastric fundus to the heterogeneous mass, both devoid of contrast enhancement. **(D)** Intracavitary contrast injection confirmed appropriate catheter tip placement distal to the fistula.

**Figure 2:**
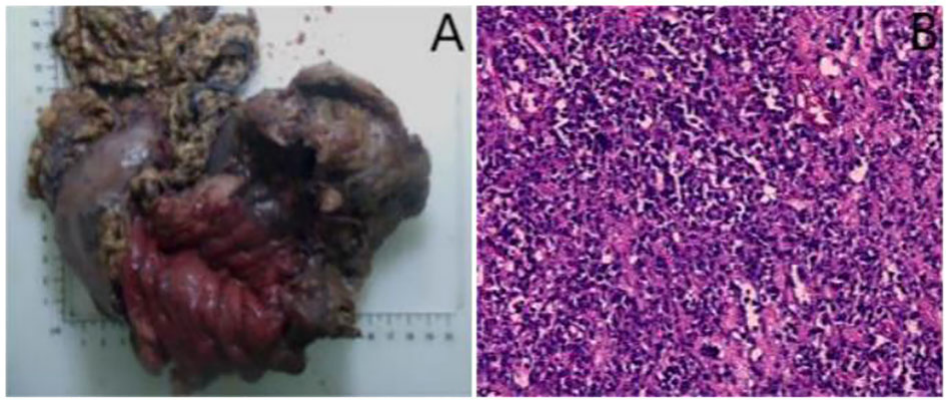
**(A)** The entire stomach, body and tail of pancreas, and spleen were resected during the surgery. **(B)** Postoperative pathology (hematoxylin-eosin staining) confirmed diffuse large B-cell lymphoma in the stomach (×100).

An 18-year-old male presented with gastric distension and epigastric pain, and was diagnosed with gastric non-Hodgkin lymphoma (diffuse large B-cell lymphoma) at an external hospital. He was subsequently referred to our institution for further management. After completing four cycles of standardized chemotherapy, the patient experienced acute hematemesis of approximately 1000 ml following the ingestion of bread and milk. No accompanying symptoms such as fever, chills, acid reflux, heartburn, abdominal bloating, or diarrhea were reported. Emergency digital subtraction angiography identified suspected bleeding (from the left gastric artery, splenic artery, and left phrenic artery), all of which were successfully embolized using gelatin sponge particles. Five days post-embolization, the patient developed sudden-onset fever and syncope. Contrast-enhanced CT scan (**Figure 1A**) revealed an abnormal density lesion between the gastric fundus and spleen, indicative of gastric perforation with adjacent exudation and localized abscess formation. A multidisciplinary team evaluation noted markedly elevated inflammatory markers and poor general condition, deeming the patient unsuitable for immediate surgery. He was transferred to our department for alternative treatment. B-mode ultrasound (**Figure 1B**) revealed heterogeneous echogenicity within the spleen, multiple hypoechoic areas, and a large irregular heterogeneous mass adjacent to the splenic hilum containing fluid components (scattered weak echoes) and extensive gas-like hyperechoic foci. After injecting 2.4 ml of contrast agent (SonoVue^®^) via the antecubital vein followed by a 5.0 ml flush of 0.9% saline, contrast-enhanced ultrasound (**Figure 1C**) revealed a large non-enhancement area in the inferolateral spleen during the arterial phase and a fistulous tract connecting the gastric fundus to the heterogeneous mass, both devoid of contrast enhancement. CEUS further delineated a viable percutaneous access route through the necrotic splenic region. We percutaneously placed an 8F drainage catheter through the splenic infarct zone, effectively avoiding injury to adjacent organs and tissues such as the intestines and pancreas. Ultrasound-guided percutaneous catheter drainage of the gastrosplenic fistula was successfully performed. After preparing a 1:100 dilution of contrast agent (1 ml of stock solution in 100 ml saline), 20 ml of the diluted contrast suspension was administered for intracavitary contrast-enhanced ultrasound. The imaging confirmed optimal position of the distal end of the drainage catheter within the fistula tract (**Figure 1D**). Dark-brown turbid fluid was drained intraoperatively, and the patient stabilized postoperatively. Follow-up contrast-enhanced CT at 20 days post-drainage showed a reduced encapsulated fluid and gas collection at the fistula site. After one month of clinical improvement, the patient remained in stable condition and underwent total gastrectomy with distal pancreatectomy and splenectomy via exploratory laparotomy. Postoperative pathology confirmed diffuse large B-cell lymphoma (**Figures 2A, B**). The patient was discharged and continued maintenance chemotherapy for non-Hodgkin lymphoma. At 13 months postoperatively, he remains in good general condition with stable vital signs.

## 3 Discussion

Gastrosplenic fistula is an exceptionally rare but serious complication that can arise from both malignant and benign conditions, most commonly linked to aggressive lymphomas involving the spleen and gastric wall. Diffuse large B-cell lymphoma represents the predominant histological subtype, accounting for approximately 85% of reported cases. Interestingly, gastrosplenic fistulas may develop either during lymphoma treatment or serve as the initial manifestation of the underlying malignancy (1, 3). The close anatomical relationship between the stomach and spleen, reinforced by the gastrosplenic ligament, creates a predisposed environment for fistula formation. This pathological connection is often driven by tumor infiltration, infectious erosion, or tissue necrosis (3–5).

Clinically, gastrosplenic fistula presents with nonspecific symptoms and may remain asymptomatic initially. When symptomatic, patients commonly report abdominal discomfort, signs of infection, dyspepsia, hematemesis, melena, or syncope (6). Left upper abdominal pain is the most frequently reported initial symptom (7). However, acute upper gastrointestinal bleeding secondary to gastrosplenic fistula can be life-threatening, necessitating urgent embolization or surgical intervention to prevent fatal outcomes (8–10). Therefore, clinicians should maintain a high index of suspicion for gastrosplenic fistula in patients with gastric or splenic lymphoma who present with upper gastrointestinal bleeding (4).

Diagnosing gastrosplenic fistula is challenging due to the nonspecific nature of symptoms. CT has been shown to be more sensitive than endoscopy or upper gastrointestinal imaging in detecting these fistulas (7). On unenhanced CT scans, the presence of a gas-fluid level within the spleen should raise suspicion. Contrast-enhanced CT is considered the diagnostic modality of choice, with visualization of a contrast-filled channel between the stomach and spleen being a specific indicator of the condition (5, 11). Ultrasound offers a radiation-free, readily accessible, and real-time imaging alternative. It can rapidly detect perisplenic fluid collections, abscesses, or splenic parenchymal abnormalities such as infarction secondary to gastrosplenic fistula. CEUS, using intravenous microbubble contrast agents like sulfur hexafluoride, dynamically visualizes microvascular perfusion. In cases where the fistula is associated with a splenic abscess, CEUS distinctly demonstrates enhancement of the abscess wall with internal non-perfused areas and minimize the risk of inadvertent injury to the adjacent organ (12). Additionally, intraluminal administration of contrast can reveal abnormal flow toward the gastric lumen and the spleen, definitively confirming the presence of a fistulous tract.

Both surgical and non-surgical approaches have been employed in the management of gastrosplenic fistula, a rare and serious complication. Surgical resection remains the mainstay of treatment and typically involves splenectomy, along with partial or total gastrectomy and, in some cases, resection of adjacent organs (8). While non-surgical options such as chemotherapy have been reported, these may increase the risks of bleeding, perforation, infection, and mortality (13, 14). Preoperative splenic artery embolization has proven effective in stabilizing patients with hemodynamic compromise due to massive hematemesis, offering a means to improve their condition before definitive surgical intervention (15, 16). Although surgery is generally regarded as the most definitive treatment, the overall prognosis for patients with gastrosplenic fistula secondary to lymphoma remains poor (1), particularly in those who are critically ill or unwilling to undergo surgery.

In such high-risk cases, there is an urgent need for alternative strategies that either improve the patient’s condition sufficiently for surgery or serve as viable non-surgical interventions. Based on the study by Aribas et al. (17), conspicuous radiolucent fistulous tract has been detected by giving contrast medium via the catheter on CT-cystography; subsequently splenectomy and fistula resection after abscess catheter placement. In this case, we first utilized B-mode ultrasound followed by CEUS to identify avascular zones and areas devoid of vital structures, thereby guiding safe and accurate percutaneous drainage catheter placement. Ultrasound-guided percutaneous drainage is relatively straightforward, minimally invasive, time-efficient, and associated with fewer complications. It represents a valuable therapeutic option, especially in patients with localized effusions or abscesses who are poor surgical candidates. CEUS, in particular, allows for real-time visualization and precise delineation of vascular anatomy, enabling the operator to identify a puncture trajectory that avoids major vessels, nerves, intestinal loops, and other critical structures. In the present case, this approach enabled successful percutaneous drainage of the fistula-associated abscess, providing both symptomatic relief and infection control. Although reports of managing gastrosplenic fistula using this method remain scarce, our experience suggests that this combined ultrasound-guided strategy could serve as an effective adjunct or bridging therapy in critically ill patients, buying crucial time before definitive surgical intervention. Moreover, in patients with smaller fistulous tracts, we cautiously speculate that external drainage combined with nasogastric-jejunal tube placement may promote spontaneous closure of the fistula, potentially avoiding the need for surgery altogether. This minimally invasive strategy may offer a promising avenue for future clinical application and warrants further investigation.

## 4 Conclusion

Gastrosplenic fistula is a rare and potentially life-threatening complication of lymphoma, which may develop spontaneously or as a result of chemotherapy. Early diagnosis and timely intervention are essential for improving prognosis. In this case, intravenous CEUS was utilized to assess the spatial relationship between the lesion and adjacent anatomical structures, thereby facilitating the selection of an optimal and safe puncture trajectory. Following successful ultrasound-guided placement of a drainage catheter, intracavitary CEUS was performed to dynamically monitor microbubble flow within the fistula. This technique enabled precise localization of the fistulous tract, including minute fistula orifices that would have been undetectable by conventional ultrasound, and also confirmed the accurate positioning of the catheter tip, preventing placement errors such as overly superficial or excessively deep insertion (18). In conclusion, although surgery remains the definitive treatment for gastrosplenic fistula, our minimally invasive approach combining CEUS with ultrasound-guided percutaneous drainage demonstrates potential as an effective adjunctive strategy. This technique may serve as a critical bridging therapy for critically ill patients awaiting surgery and offers valuable insights into the management of this rare and complex condition. Furthermore, in select patients with small fistula orifices, it may represent a less invasive alternative to surgery, warranting further clinical investigation.

## Data Availability

The original contributions presented in the study are included in the article/supplementary material. Further inquiries can be directed to the corresponding authors.
